# Genomic and neurobiological bases of variation in fighting strategies in gamecocks

**DOI:** 10.1093/molbev/msag007

**Published:** 2026-01-09

**Authors:** Tsuyoshi Shimmura, Takuma Kurachi, Yuki Matsuda, Nima Rafati, Kohei Shimura, Tatsuhiko Goto, Shin-Ichi Kawakami, Rikuto Maeda, Yohei Yamada, Mats E Pettersson, Yoshiaki Nakamura, Yuki Higashiura, Nonoko N Shimura, Andres Bendesky, Masaoki Tsudzuki, Leif Andersson

**Affiliations:** Department of Biological Production, Tokyo University of Agriculture and Technology, Fuchu, Tokyo 183-8509, Japan; Research Area of Informatics for Human-Animal Interaction, Advanced Research Center for One Welfare, Tokyo University of Agriculture and Technology, Fuchu, Tokyo 183-8509, Japan; Department of Biological Production, Tokyo University of Agriculture and Technology, Fuchu, Tokyo 183-8509, Japan; Department of Biological Production, Tokyo University of Agriculture and Technology, Fuchu, Tokyo 183-8509, Japan; Science for Life Laboratory, Department of Medical Biochemistry and Microbiology, Uppsala University, Uppsala SE75123, Sweden; Department of Biological Production, Tokyo University of Agriculture and Technology, Fuchu, Tokyo 183-8509, Japan; Research Center for Global Agromedicine, Obihiro University of Agriculture and Veterinary Medicine, Obihiro Hokkaido 080-8555, Japan; Graduate School of Integrated Sciences for Life, Hiroshima University, Higashi-Hiroshima, Hiroshima 739-8528, Japan; Department of Biological Production, Tokyo University of Agriculture and Technology, Fuchu, Tokyo 183-8509, Japan; Department of Biological Production, Tokyo University of Agriculture and Technology, Fuchu, Tokyo 183-8509, Japan; Science for Life Laboratory, Department of Medical Biochemistry and Microbiology, Uppsala University, Uppsala SE75123, Sweden; Graduate School of Integrated Sciences for Life, Hiroshima University, Higashi-Hiroshima, Hiroshima 739-8528, Japan; Department of Biological Production, Tokyo University of Agriculture and Technology, Fuchu, Tokyo 183-8509, Japan; Department of Biological Production, Tokyo University of Agriculture and Technology, Fuchu, Tokyo 183-8509, Japan; Zuckerman Mind Brain Behavior Institute, Columbia University, New York, NY 10027, USA; Department of Ecology Evolution and Environmental Biology, Columbia University, New York, NY 10027, USA; Graduate School of Integrated Sciences for Life, Hiroshima University, Higashi-Hiroshima, Hiroshima 739-8528, Japan; Science for Life Laboratory, Department of Medical Biochemistry and Microbiology, Uppsala University, Uppsala SE75123, Sweden; Department of Veterinary Integrative Biosciences, Texas A&M University, College Station, TX 77843, USA

**Keywords:** aggressive behavior, fighting strategy, chicken, bird, population genomics

## Abstract

Aggression is an essential animal behavior for survival, particularly in situations where fighting cannot be avoided. In such situations, the choice of fighting strategy (eg biting, charging, or defending) is critical. Although the molecular bases of fighting and aggressiveness have been previously studied, how genetic, transcriptional, and neurobiological mechanisms contribute to the choice of fighting strategy remains largely unknown. Here, we use two subpopulations of chickens bred for cockfighting that show markedly different fighting strategies: offensive and defensive attack. A genome-wide screen comparing individuals from the two subpopulations indicated a polygenic background and we identified 15 candidate genes, five of which are implicated in neuronal development. Among these, the transcription factor gene *FOXP1* was notable. *FOXP1* is essential for neuronal development in the brain and has been implicated in the regulation of motor circuits. Transcriptomic analysis of the diencephalon also revealed differential expressions of genes involved in neurodevelopment, as well as in the synthesis and release of neurotransmitters. RNA-sequencing and immunohistochemistry suggested that activation of the indirect pathway of the brain motor circuit promotes the defensive fighting strategy. This was further supported by behavioral pharmacological experiments targeting dopaminergic signaling. Taken together, our results indicate that genomic variation and altered expression of neurodevelopment-related genes underlie differences in fighting strategies, and that the neuroendocrine changes in brain circuits further modulate these behavioral outcomes.

## Introduction

Classic game theory, particularly the hawk–dove game (also known as the game of chicken) by John Maynard Smith, has proposed the existence of evolutionary stable strategies involving individuals that behave aggressively (“hawks”) and those that avoid fights or defend themselves (“doves”) (Smith 1964, 1988; Smith and Price 1973): When all individuals in a population adopt the hawk strategy, aggression escalates, making the strategy evolutionary unstable. On the other hand, when all individuals adopt the dove strategy, fighting is not observed, but this situation is also not evolutionarily stable because doves flee when new mutations that lead to hawks invade the population. Therefore, a mixed strategy involving both hawks and doves makes the population evolutionarily stable. Game theory also implies the existence of genetic variants that shape fighting strategies. However, empirical support for the classic game theory scenarios is lacking.

Aggression is an essential behavior for the survival of individuals, and animals fight for limited food, mate, and territory (Archer 1988). When animals cannot avoid fighting, the strategies they choose can be critical for survival. In social behaviors, aggressive behavior has been intensively studied, and the molecular bases of fighting and aggressiveness have been previously explored. For example, genetic studies have identified mutations underlying extensively high aggressiveness in humans (Brunner et al. 1993) and domestic animals (Bendesky et al. 2023); transcriptomic analyses of brain tissue have revealed gene expression changes associated with aggression in bees (Whitfield et al. 2003), fishes (Renn et al. 2008; Sanogo and Bell 2016), and birds (Levy et al. 2025); and neurobiological studies have elucidated neural mechanism for the switching of aggression in mice (Nelson and Trainor 2007). However, the genetic bases of fighting strategies—how genetic differences affect gene expression in the brain, and through which neuronal circuits—remain largely unknown. To characterize the genetic bases of fighting strategies, it is important to identify individuals or populations showing different fighting strategies for comparative analysis.

**Figure 1:**
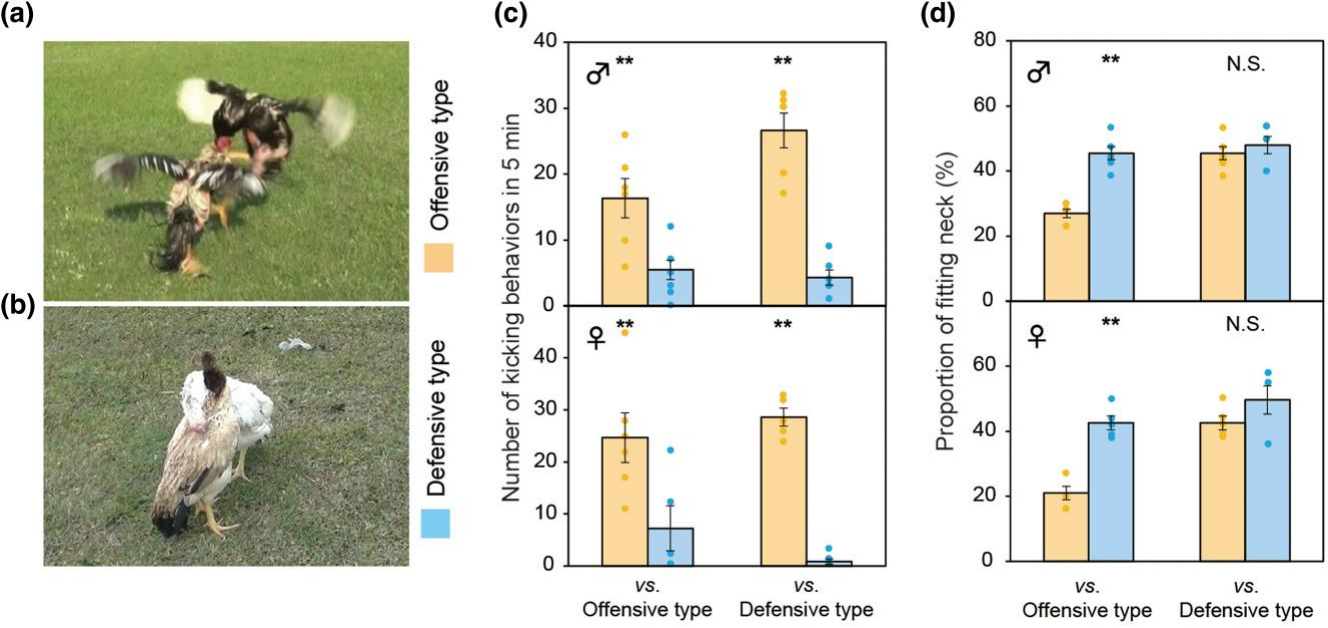
Type-specific fighting strategies of offensive and defensive types independent of sex and opponent type. a, b) The offensive type keeps jumping and kicking each other a), but the defensive type keeps holding their own neck around the opponent's neck to defend the attack b). c, d) Comparison of fighting strategies of the offensive and defensive types when meeting the same or opposite type in the behavioral test for 5 min. c) In both males and females, the offensive type showed the typical kicking behavior more than the defensive type regardless of when they fought against the offensive or defensive types (**P < 0.01, *t*-test; mean ± SEM, *N* = 5–6). d) The defensive type spent more time on fitting the neck than the offensive type in both sexes when fought against the offensive type (**P < 0.01, *t*-test; mean ± SEM, *N* = 5–6). N.S. indicates no significant differences (*P* = 0.47 [male] and *P* = 0.21 [female]).

Jungle fowls, ancestors of chickens, were used for cockfighting as far back as the Indus Valley civilization (from 3,300 BCE to 1,300 BCE) (Sherman 2002). Fighting cocks have been domesticated for fighting purposes, while maintaining the high aggressiveness of jungle fowls, and they fight immediately when face to face (Luo et al. 2020). Fighting cocks throughout the world appear to have a common origin and share genetic variants in the gene ISPD that likely mediate their overall “gameness”—their propensity to participate in a fight (Bendesky et al. 2023). However, genetic variants that shape which fighting strategy particular gamecocks adopt are not yet known. Although general fighting strategies of domesticated chickens are pecking and kicking with jumping (Fig. 1a: offensive type), we found a subpopulation with a preference for fighting defensively in a breed intended for cockfighting (Fig. 1b: defensive type). Whereas individuals of the offensive type (UWA) keep jumping and kicking each other (Fig. 1a), defensive individuals (HIKU) keep holding their own neck around the opponent's neck to defend themselves from attack (Fig. 1b). The neck-fitting behavior, a typical behavior of the defensive type, was defined as the action of pressing the neck against the opponent, which can be quantified from video recording. Breeders have suggested that the defensive type emerged from the offensive type relatively recently, but at least 40 years ago. The defensive fighting strategy, which avoids injuries, has been preferred and closely kept by Japanese breeders. The remarkable divergence in fighting strategies between such closely related populations offers a unique opportunity to define the genomic architecture of social behavior and to identify pathways and genes that contribute to the evolution of different types of aggressive behavior (Bendesky et al. 2023).

Here, we apply an integrative approach combining population genomics, transcriptomics, and neurobiology in the two populations exhibiting distinct fighting strategies, and show that genetic variation affecting motor circuits shapes the fighting strategy employed.

## Results

### Offensive and defensive fighting strategies in fighting

We identified two subpopulations in a cockfighting breed (Oh-Shamo) that do not differ in the latency to fight, yet employ different fighting strategies. When we measured fighting strategy prevalence by a behavioral test, putting 2 individuals face to face, the offensive individuals showed their typical kicking behavior more often than the defensive type in both sexes, regardless of the type of opponent (Fig. 1c). The defensive type exhibited a high frequency of neck-fitting behavior in both sexes regardless of whether they were confronted with an offensive or another defensive type (Fig. 1d). In contrast, the offensive type showed a high level of neck-fitting only when interacting with the defensive type, as a result of the defensive type fitting their neck and maintaining close contact (Fig. 1d). Overall, these behavioral tests indicated that kicking behavior characteristic of the offensive type, as well as the holding behavior associated with the defensive type, were observed in both sexes and independent of the type of opponent.

We also confirmed that the individuals crossing among the same types showed the fighting strategies of that type and that the F1 hybrid after crossing the two types showed both types of behaviors (Fig. S1). This suggests that there is a genetic component to the fighting strategies of these gamecock subpopulations.

### Genetic architecture of fighting strategies

**Figure 2:**
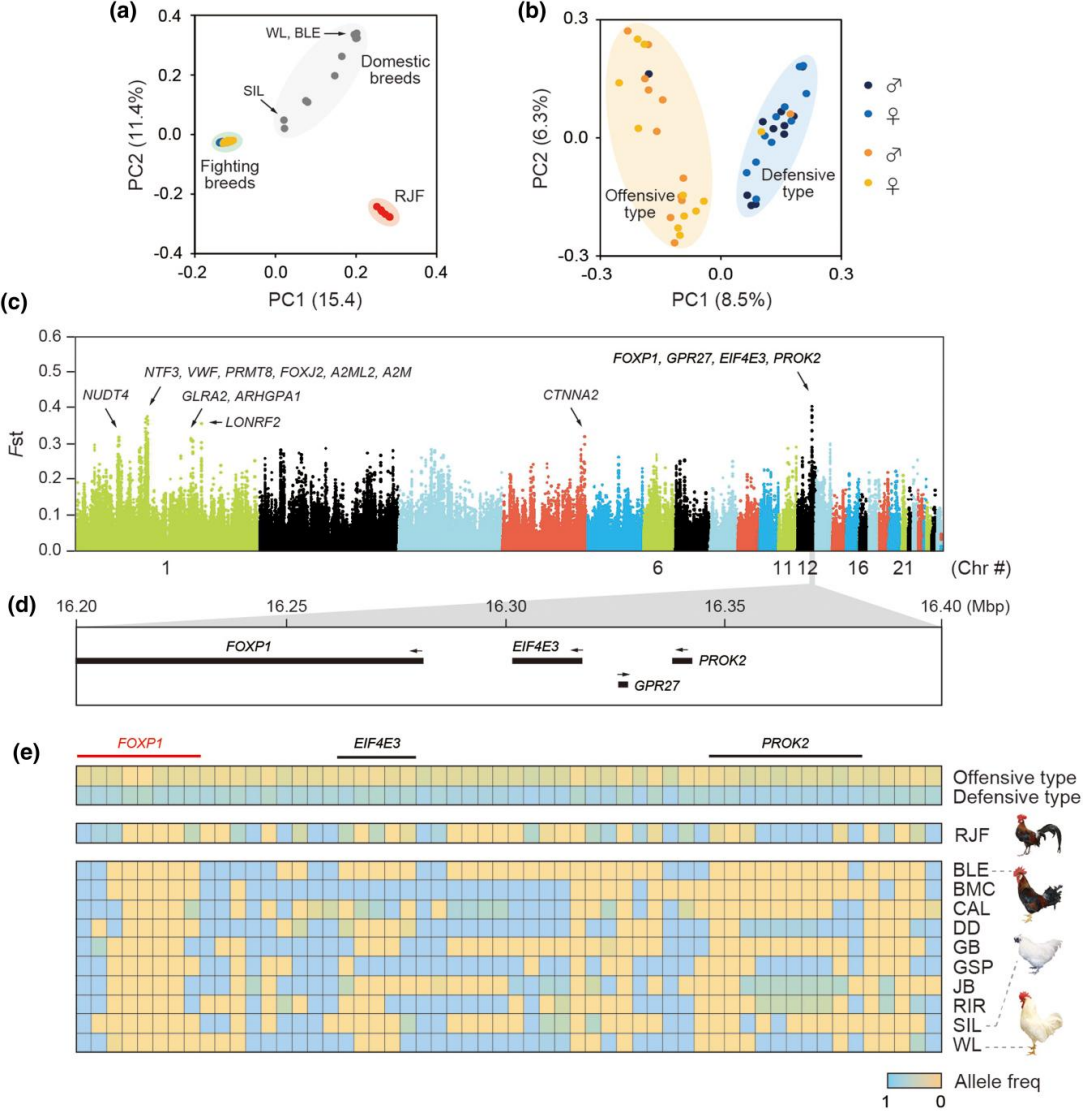
Genetic architecture of fighting strategies. a) PCA of fighting breeds, red jungle fowls, and domestic breeds (all male). b) Another PCA of offensive and defensive types in fighting breeds. Although three individuals showed atypical behavior clustered with the opposite behavioral type, the overall PCA patterns showed clear separation between offensive and defensive types, with the majority of individuals clustering appropriately according to their behavioral classification. c) Genome-wide *F_ST_* screen comparing offensive and defensive types using whole-genome sequencing of individual DNA. Candidate genes in regions with *F_st_* > 0.3 are highlighted. d) A 200-kb associated region on chromosome 12. e) Allele frequencies of SNPs of the two types of fighting breeds, red jungle fowls, and domestic breeds within the region on chromosome 12. The most common alleles in the offensive type were used as the reference allele, and therefore, the SNPs with high frequencies of the major allele in the offensive type are colored yellow, while a high frequency of the minor alleles is colored blue. *RJF,* red jungle fowl; *BLE,* brown leghorn; *CAL,* white wyandotte; *DD,* dandarlawi; *GB,* game bantam; *GSP,* fayoumi; *JB,* Japanese bantam; *RIR,* Rhode Island red; *SIL,* silky fowl; and *WL* white leghorn.

To address the genetic basis underlying the fighting strategies, we performed population genomic analysis using whole-genome sequencing. We generated approximately 7× genome coverage per individual from 23 offensive types (12 males and 11 females) and 22 defensive types (11 males and 11 females), as well as 6 red jungle fowl (all males; Table S1), and 10 domestic inbred populations kept at Nagoya University (Matsuda et al. 2025). We aligned the genomic reads against the chicken reference genome (GCA_000002315.3) for variant identification, yielding 7.5 million filter-passed single-nucleotide polymorphisms (SNPs). Principal component analysis (PCA) based on the set of SNPs showed three main clusters consisting of fighting breeds, other domestic breeds, and red jungle fowls (Fig. 2a). The offensive and defensive types in the fighting breed clustered as genetically close (Fig. 2a) but two distinct groups (Fig. 2b), a favorable situation for identifying the genomic regions that differentiate the groups by population genomics, because background divergence, including between breeding populations, can be expected to be moderate. Therefore, we performed a genome-wide scan using the fixation index (*F*_st_) between the offensive and defensive types (Fig. 2c). We identified a total of 42 regions with *F*_st_ > 0.3, in which 15 candidate genes were located. Five of these are implicated in neuronal development: *NTF3* and *PRMT8* on chromosome 1, *CTNNA2* on chromosome 4, and *FOXP1* and *PROK2* on chromosome 12 (Fig. 2c). We then focused on a region, located on chromosome 12 and including the highest overall *F*_st_ peak (Fig. 2c). This 200-kb region on chromosome 12 contained the *FOXP1* (forkhead box P1), *EIF4E3* (eukaryotic translation initiation factor 4E family member 3), *GPR27* (G-protein-coupled receptor 27), and *PROK2* (prokineticin 2) genes (Fig. 2d). An analysis using the Genome Analysis Toolkit (GATK) revealed no nonsynonymous mutations, insertions, deletions or differences in splicing in either of the four genes (Fig. S2, Table S2).

The pecking and kicking behavior of the offensive type is normal aggressive behavior commonly observed in jungle fowls and domestic chicken breeds when they determine the social ranks known as “pecking order” (Wood-Gush 1955). Therefore, we speculated that the defensive phenotype may be the derived state and that other chicken breeds were likely to have similar alleles to offensive types and that a region of mostly fixed alleles would be observed in the defensive type group. To search for such a subregion within the 200-kb region on chromosome 12, we extracted the SNPs with highly divergent allele frequencies between offensive and defensive types (Fig. 2e, Table S2) and then compared the allele frequencies of SNPs among the two types, as well as in red jungle fowls and the domestic breeds (Fig. 2e). The most striking result was that the defensive type was nearly fixed across this region (Fig. 2e), while the offensive type as well as other populations were more variable as expected if the defensive haplotype is derived.

### Imbalance in motor pathway changes fighting strategies

**Figure 3:**
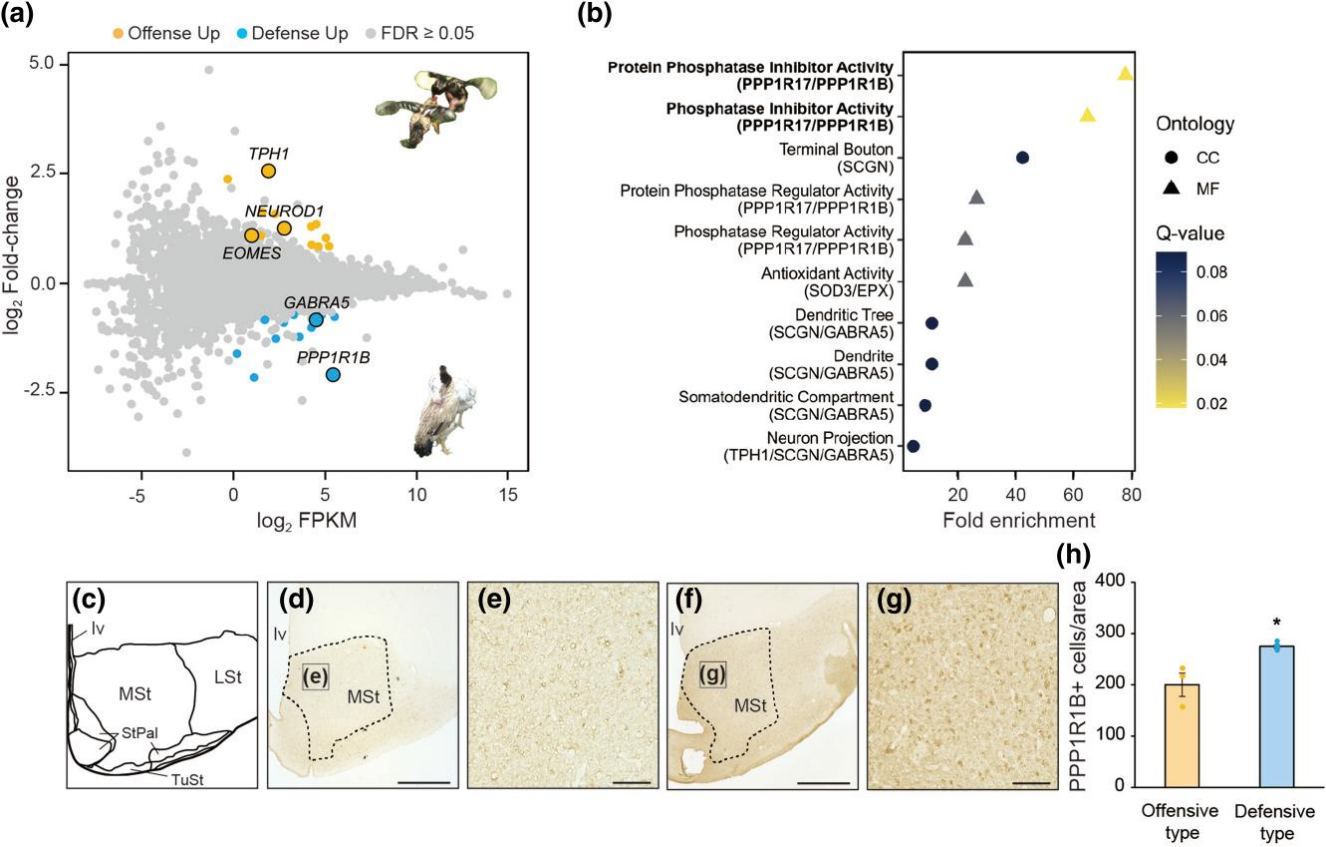
Identification of DEGs in brain associated with difference in fighting strategies. a) MA-plot of transcripts of annotated genes analyzed by RNA-sequencing. MA-plot is a visualization tool to display differences in expression levels between two groups, and the number of differently expressed transcripts is indicated by colored dots, compared between the offensive and defensive types (*P* < 0.05, *Q* < 0.05). Fragments per kilobase of exon per million mapped reads (FPKM) were calculated by the geometric mean between offensive and defensive types in each transcript. b) Top 10 enriched GO terms among DEGs. The significantly enriched terms (*Q*-value < 0.05) are shown in bold. The horizontal axis represents the rich factor (foreground gene ratio/background gene ratio). The color of each dot indicates the *Q*-value, and the shape indicates the ontology category: cellular component (CC) or molecular function (MF). Biological process (BP) terms were not included among the top ten enriched categories. c) Schematic drawing of the chicken striatum. *MSt*, medial striatum; *LSt*, lateral striatum; *StPal*, striopallidal area; *TuSt*, olfact tub; *lv*, lateral ventricle. d–g) Representative image of PPP1R1B immunoreactivity in the striatum of the offensive d and e) and defensive types f and g). Scale bar: 1,000 µm d and f) and 100 µm e and g). h) The number of PPP1R1B-positive cells was higher in the defensive type than in the offensive type (*P < 0.05, *t-test; mean ± SEM, *N* = 3–4).

To further explore the molecular basis for the variation in fighting strategies, we conducted RNA-sequencing to compare gene expression between offensive and defensive types. We focused on the brain region, including the diencephalon, subpallium, and brainstem, which encompasses several key nuclei involved in the regulation of aggressive behavior—such as the ventromedial hypothalamic nucleus (Olkowicz et al. 2016; Aleyasin et al. 2018)—and collected the region from sexually mature individuals. No candidate genes identified by the genome-wide *F*_ST_ screen showed differential expression (Table S3; eg *FOXP1* expression: log_2_FC = 0.1, log_2_FPKM = 2.6). However, the comparison identified 27 annotated genes with *Q*-value (false discovery rate [FDR]-adjusted *P*-value) < 0.05 (Fig. 3a). Among them, two genes (*NEUROD1* and *EOMES*) are involved in neurodevelopment and three genes (*TPH1*, *PPP1R1B*, and *GABRA5*) are associated with the synthesis and release of neurotransmitter (Fig. 3a). Notably, four genes (*TPH1*, *EPX*, *PPP1R1B*, and *TMEM61*) met both criteria of a *Q*-value of < 0.05 and a large expression difference (|fold-change| > 4). The gene set enrichment analysis of gene ontology (GO) also revealed that *PPP1R1B* and *PPP1R17* were associated with phosphatase-related molecular function (Fig. 3b, Table S4). Among these, *PPP1R1B*, which was upregulated in the defensive type, has previously been reported to influence behavioral change and be under the control of *FOXP1* (Precious et al. 2016; Anderson et al. 2020).

PPP1R1B (protein phosphatase 1 regulatory inhibitor subunit 1B), also known as DARPP32 (dopamine and cAMP-regulated neuronal phosphoprotein 32), is a phosphoprotein that controls the signaling in dopaminergic neurons and is highly expressed in the striatum in basal ganglia that receive the projection of dopaminergic neurons (Hemmings et al. 1992; Anderson et al. 2020). Our spatial analysis, using immunohistochemistry, identified expression of PPP1R1B in the striatum, as expected, and revealed higher expression in the defensive type than in the offensive type (Figs. 3c–h and S3).

**Figure 4:**
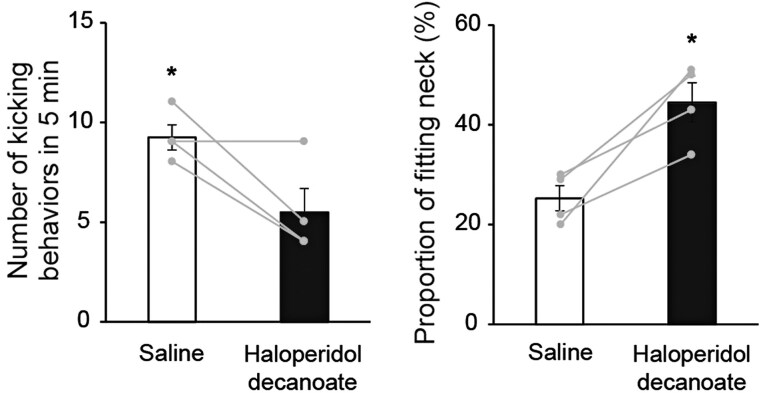
Activation of the indirect pathway by intramuscular administration of haloperidol decanoate induces defensive attack. Kicking behavior was significantly decreased while fitting behavior was increased in the offensive type that received haloperidol decanoate compared with saline (*P < 0.05, *t-test; mean ± SEM, *N* = 4).

The indirect pathway of the striatum, inhibiting movement, expresses the dopamine D2 receptor (Anderson et al. 2020). Haloperidol is used as an antipsychotic drug in humans, acting as an inverse agonist of D2 receptors and activating the indirect pathway (Beresford and Ward 1987; Matsunaga et al. 1987). When we administered haloperidol intramuscularly to offensive-type chickens, they showed significantly reduced offensive-type-specific kicking behavior and increased defensive-type-specific fitting behavior compared with the offensive type that received saline (Figs. 4a and S3a, b). The *PPP1R1B* expression in the diencephalon was also higher in the offensive type that received haloperidol, supporting the notion that the drug successfully activated the indirect pathway in the brain (Fig. S3c). Taken together, these results strongly suggest that an imbalance of brain motor circuits results in different fighting strategies. More specifically, defensive behavior is induced by activation of indirect pathways in the striatum (Fig. S6).

## Discussion

In this study, using a unique chicken population in which fighting strategies largely differed among individuals, we explored the genetic basis of fighting strategies and its functional mechanism. Population genomics using genome-wide *F*_ST_ screen comparing offensive and defensive types indicated that differences in fighting strategy has a polygenic basis (Fig. 2c). We identified 15 candidate genes and five of these have been previously associated with neuronal development in the brain, including neurotrophin 3 (*NTF3*) (Tessarollo et al. 1994), protein arginine methyltransferase 8 (*PRMT8*) (Kim et al. 2015), catenin alpha 2 (*CTNNA2*) (Schaffer et al. 2018), forkhead box P1 (*FOXP1*) (Bacon and Rappold 2012; Anderson et al. 2020), and prokineticin 2 (*PROK2*) (Traboulsi et al. 2015). None of these candidate genes showed differential expression in the brain between sexually mature offensive and defensive types (Table S3; eg *FOXP1* expression: log_2_FC = 0.1, log_2_FPKM = 2.6). This might be due to (i) the role of these genes being restricted to earlier developmental stages (Bacon et al. 2015), (ii) its expression being localized to specific brain areas such as the striatum (Anderson et al. 2020) not included in the present study, (iii) cell type-specific expression patterns being masked in bulk tissue analysis (Araujo et al. 2015), (iv) nontranscriptional regulatory mechanisms, or (v) limited sample size (4 biological replicates in this study), which may have led to false negatives (Conesa et al. 2016; Schurch et al. 2016). On the other hand, our transcriptomic analysis detected differential expression of genes involved in neurodevelopment, including neuronal differentiation 1 (*NEUROD1*) (Tutukova et al. 2021) and eomesodermin (*EOMES*; also known as T-box brain protein 2 [TBR2]) (Lv et al. 2019). Both of these genes were downregulated in the defensive type (Fig. 3a), suggesting impaired neuronal development and neurocircuit formation in these individuals. Furthermore, RNA-sequencing identified differentially expressed genes (DEGs) involved in synthesis and release of neurotransmitters: tryptophan hydroxylase 1 (*TPH1*), involved in serotonin biosynthesis; protein phosphatase 1 regulatory subunit 1B (*PPP1R1B*/*DARPP-32*), a key component of dopamine signaling; and gamma-aminobutyric acid type A receptor subunit alpha5 (*GABRA5*), which is involved in GABAergic inhibitory neurotransmission. Although our RNA-sequencing data alone cannot predict the directionality of aggression, previous studies suggest that downregulation of TPH1/2 promotes aggressive behavior (Strekalova et al. 2023), upregulation of PPP1R1B enhances aggression (Reuter et al. 2009), and upregulation of GABRA5 inhibits it (Agrawal and Dwivedi 2020). These findings imply that interactions among serotonergic (TPH1/2), dopaminergic (PPP1R1B), and GABAergic (GABRA5) neurotransmission systems jointly regulate aggressive behavior (Aleyasin et al. 2018). Taken together, these genomic and transcriptomic profiling findings suggest that genomic variation and altered expression of neurodevelopment-related genes underlie differences in attack strategies, and that the neuroendocrine changes in brain function further modulate these behavioral outcomes.

Among the integrative results of population genomics (Fig. 2) and transcriptomic analyses (Fig. 3), the most compelling hypothesis to emerge is that polymorphisms in neurodevelopment-related genes may lead to imbalanced motor pathways, thereby altering fighting strategies. One of the most promising positional candidate genes identified is *FOXP1*, a transcription factor belonging to subfamily P of the forkhead box (FOX) family. *FOXP1* is known to play an important role in neuronal development in the brain, and mutations have been reported to cause motor and cognitive impairment in humans and mice (Bacon and Rappold 2012; Anderson et al. 2020). Mice heterozygous for a *FOXP1* null mutation also showed a change in social behavior (Araujo et al. 2015). Genetic variation in *FOXP1* between offensive types and defensive types may influence behavior at least in part through the regulation of its downstream target gene, *PPP1R1B.* Notably, we found that *PPP1R1B* is expressed 4.2-fold more in the diencephalon of defense types than in offensive types. Furthermore, activating the dopamine receptor 2-PPP1R1B signaling pathway in the offensive type promoted defensive behavior, supporting a causal link between this molecular pathway and divergent aggression strategies.

The movement of animals is controlled by two circuits in the striatum: a direct pathway facilitating movement and an indirect pathway inhibiting movement (Figure S6) (Gerfen and Surmeier 2011). The FOXP1 and PPP1R1B proteins are co-localized in SPNs of the brain striatum, and the development of PPP1R1B-positive SPNs is under the control of FOXP1 (Precious et al. 2016). FOXP1 is also crucial for maintaining the cellular composition of the striatum, especially indirect pathway SPNs (iSPNs) where PPP1R1B is expressed (Anderson et al. 2020). In fact, when *FOXP1* was specifically knocked out in iSPN, abnormal motor learning was observed. The *FOXP1* heterozygous mice also showed a change in social behavior due to increased excitability of iSPNs (Araujo et al. 2015). Although further experimental work is needed to explore the possible role of FOXP1 in regulating fighting behavior in chickens, our functional genomics indicates at least the imbalance of brain motor circuits by genetic polymorphisms in neurodevelopment-related genes changes fighting strategies.

Although the difference in fighting strategies may be particularly evident under social conditions that require active engagement in conflict, the results from a behavioral test involving jumping onto an elevated perch showed that defensive-type chickens were capable of reaching similar heights as offensive types (Fig. S4). This suggests that defensive-type chickens adjust their fighting strategy depending on the social context. Recent studies have reported the relationship between higher cerebral function and movement: coordination on a time scale of tens of milliseconds between hippocampal cognitive representation and motor processes (Joshi et al. 2023) and the somato-cognitive action network on the motor cortex for integrating goal and movement (Gordon et al. 2023). PPP1R1B, showing differential expression between offensive and defensive types in this study, is a molecule that integrates dopamine and other neurotransmitters such as serotonin and glutamate in the striatum and is a controller for the selection of movement and learning (Svenningsson et al. 2004; Nishi et al. 2005). Therefore, an interesting follow-up question is how the genetic variation in the genes we identified is involved in signal processing from higher brain functions and why changes in fighting strategies have emerged.

Sixty years ago, John Maynard Smith proposed game theory predictions for the existence of evolutionary stable strategies involving individuals that behave aggressively and those that avoid fights or defend themselves (Archer 1988). Using novel molecular technologies, we have now explored the genetic architecture, molecular bases, and affected neuronal circuits of these different fighting strategies. Our genomic dissection of fighting strategies—from behavior to candidate gene—provides an insight into how complex social behavior patterns evolve and provides a first empirical foundation for the classic game theory scenarios.

## Materials and methods

### Ethic statement

Animals were treated in accordance with the guidelines of Hiroshima University. All experimental protocols were approved by the Animal Experiment Committee of Hiroshima University (C22-35), Japan.

### Animals and behavioral test

The animals included in this study were collected from three different private farms that keep 50–100 birds each. The mature fighting cocks kept in individual cages were used for assessing fighting strategies. The two individuals from the same sex were faced to fight in an open field with different pairs: both offensive types, offensive and defensive types, or both defensive types (*N* = 5–6 per type). The behaviors were recorded for 5 min using a video camera (FDR-AX30, Sony). No injuries were observed in any of the individuals during the behavioral test. The kicking behavior and neck-fitting behavior of all individuals were scored based on predefined definitions by observing each video twice per individual. For the kicking behavior, which is typical of the offensive types, one kicking behavior was defined as a single kick performed during each jump, and the number of kicks was counted over a 5-min observation period. For the neck-fitting behavior, which is typical of the defensive type, the recorded movie was divided into still images at 5-s intervals using GOM player, and the number of fitting necks was quantified by scan sampling. In this case, both individuals were considered to be involved in each neck-fitting event, so the number of events was identical for the 2 individuals involved in each interaction.

Individuals from the offensive and defensive types at 8 weeks of age reared in floor rearing were used for assessing motor ability under normal conditions for 12 days (*N* = 7 per group). The wooden perch (4 cm deep × 3 cm height) was equipped at a height of 25 cm and then elevated gradually (50, 60, 80, and 100 cm). The maximum height of perch that individual chickens perched on was measured.

In the behavioral pharmacology test (Fig. 4), mature male offensive-type chickens received an intramuscular administration of 1 ml of haloperidol decanoate (100 mg/ml) or saline (*N* = 4 per group). Each chicken was subjected to both treatments in a randomized order, with a 2-month interval between treatments, as the effects of haloperidol on D2 receptors wear off over this period. The behavioral test, in which they were faced with the defensive type, was performed 14 d after the administration, and the number of kicking behaviors was compared between treatments. In the supporting experiment (Fig. S3), the number of kicking behaviors was compared before and 3 d after haloperidol administration. Different individuals were used for the experiments shown in Figs. 4 and S3.

### DNA samples and whole-genome sequencing analysis

Blood samples of fighting cocks were obtained after obtaining informed consent from three breeders in Japan (*N* = 22–23 per type, Table S1). Blood samples of red jungle fowls were obtained from the Avian Bioscience Research Center, Nagoya University, Japan (Table S1). Blood samples were collected from the wing vein with heparin as an anticoagulant and stored at −20 °C until DNA extraction. DNA was extracted using the DNeasy blood and tissue kit (QIAGEN). The quantity and quality of the DNA samples were evaluated using a Qubit fluorometer and agarose gel electrophoresis.

Libraries from each DNA sample were sequenced with 2 × 150 bp paired-end reads in an Illumina HiSeqX sequencer to a median depth of 6.5× for individual samples (Table S1). Together with 10 inbred chickens sequenced in the previous study (Matsuda et al. 2025), the sequenced reads were quality checked using FastQC (https://www.bioinformatics.babraham.ac.uk/projects/fastqc/) and then aligned to the chicken reference genome (Gallus_gallus-5.0, GCA_000002315.3) using Burrows-Wheeler Aligner (BWA; version 0.7.15) (Li and Durbin 2009) with default parameters. The alignments were further checked for PCR duplicates using PICARD (http://picard.sourceforge.net/). The Genome Analysis Toolkit (GATK) was used for base quality recalibrations, insertion/deletion (INDEL) realignment, SNP and INDEL discovery, and genotyping according to GATK best practice recommendations (McKenna et al. 2010; DePristo et al. 2011; Van der Auwera et al. 2013). Low-quality SNP calls were filtered out by the following thresholds: SNP quality (QUAL < 100.0); quality by depth (QD < 2.0); strand bias (FS > 30.0); mapping quality (MQ < 40.0); rank sum of base quality (|BaseQRankSum| > 2.0); rank sum of clipped bases (|ClippingQRankSum| > 2.0); rank sum of mapping quality (|MQRankSum| > 2.0); and rank sum of read position bias (|ReadPosRankSum| > 2.0). Thresholds were chosen on the basis of the distribution of each of these parameters from the raw variant calls. The threshold-passed SNPs were used for the downstream analysis.

To estimate the genetic distance, genetic principal component analysis (PCA) was done using PLINK (version 1.9) (Purcell et al. 2007).

To screen the genetic signature of selection for fighting strategies genome-widely, the genetic divergence (*F*_st_) between the offensive and defensive types was calculated using the sequence data of individual DNA samples by VCFtools (version 0.1.13) (Danecek et al. 2011) by a 10-kb genomic window with 5-kb overlap. SNPs were annotated using SnpEff (version 3.4) (Cingolani et al. 2012).

### RNA samples and genome-wide gene expression analysis

A total of eight brain samples of mature male fighting cocks, comprising four samples from each of the two types (offensive and defensive types), were collected after euthanasia by decapitation. After removing the whole brain from each chicken, the left and right telencephalons, left and right optic tectum, and cerebellum (Olkowicz et al. 2016) were manually dissected on ice using fine surgical scissors and tweezers. The remaining unsectioned part of the brain containing the diencephalon, subpallium, and brainstem (Olkowicz et al. 2016) was immediately flash-frozen on dry ice, and stored at −80 °C until RNA extraction. Total RNA was prepared using the RNeasy Lipid Tissue Mini Kit (QIAGEN). The quantity and quality of the RNA samples were evaluated using an Agilent 2100 Bioanalyzer.

Libraries from each RNA sample were sequenced with 2 × 100 bp paired-end reads in an Illumina HiSeqX sequencer at a depth of 50 million reads. The sequence reads were quality checked using FastQC and aligned to the chicken reference genome using HISAT2 (version 2.1.0) (Kim et al. 2019). The DEGs were identified using Cuffdiff in Cufflinks (version 2.2.1) (Trapnell et al. 2013) with default parameters. We defined the genes with *Q* value (FDR-adjusted *P*-value) < 0.05 as DEGs. To detect the functional enrichment of DEGs, gene set enrichment analysis of gene ontology was performed using clusterProfiler (Yu et al. 2012). We used genes expressed in our RNA-seq samples as background gene sets. The GO terms with a *Q*-value lower than 0.05 were defined as the significantly enriched terms.

### Immunohistochemistry

After deep anesthesia with inhalation of isoflurane, individuals from the offensive and defensive types (*N* = 3–4 per type) were perfused transcardially with 0.05 M phosphate-buffered saline (PBS, pH 7.8) and then 4% paraformaldehyde (PFA) in 0.05 M phosphate buffer (PB, pH 7.8). The whole brain was removed from each chicken, immersed in the same fixative at room temperature overnight, followed by cryoprotection in 30% sucrose in 0.05 M PB until they sank, embedded in OCT compound (Sakura Finetek, Japan), and stored at −20 °C until sectioning. The unsectioned whole brains were sectioned coronally on a cryostat (CM1860, Leica Biosystems) at 20 μm according to the chick brain atlas (Puelles et al. 2018). Every 6th section was mounted on an MAS-coated glass slide and processed for immunohistochemistry (IHC).

Mst extends from +7.56 to +5.20, adjacent to the lateral ventricle. The area anterior to interaural +4.72 contains the corticoseptomesencephalic tract (csm), one of the major white matter tracts. Because DARPP-32 (PPP1R1B)-ir neurons in MSt gradually decreased in the rostrocaudal direction (Schnabel et al. 1997), brain sections at interaural +6.64 mm were used for IHC. Brain sections were washed with 0.05 M PBS for 5 min, permeabilized with 0.4% Triton X-100 in 0.05 M PBS for 20 min, incubated with 0.3% H_2_O_2_ in methanol for 20 min, washed with 0.05 M PBS 3 times for 5 min each, blocked with normal goat serum (Vectastain Elite ABC Rabbit IgG Kit, PK-6101, Vector Laboratories) in 0.05 M PBS for 20 min, and incubated at 4 °C overnight with rabbit primary antibody against DARPP-32 (NB300-304, Novus Biologicals) at a dilution of 1/1,000. The sections were then washed with 0.05 M PBS three times for 5 min each, incubated with biotin-conjugated goat antirabbit secondary antibody (Vectastain Elite ABC Rabbit IgG Kit, PK-6101, Vector Laboratories) for 30 min, washed with 0.05 M PBS three times for 5 min each, incubated with Elite ABC peroxidase reagent (Vectastain Elite ABC Rabbit IgG Kit, PK-6101, Vector Laboratories) for 30 min, washed with 0.05 M PBS three times for 5 min each, incubated with 3,3′-diaminobenzidine (DAB) solution (DAB Substrate Kit, 003836, Bioenno Tech) for 195 s, and washed with distilled water twice for 5 min each.

Color photomicrographs were taken using an inverted microscope (Keyence BZ-X810) with 4×, 10×, and 20× objectives. The brightness of color photographs was adjusted to 1/150 s, 1/120 s, and 1/40 s using software (BZ-X800 Analyzer, Keyence). The monochrome photomicrographs were taken with a 20× objective and the brightness of the photographs was adjusted to 1/2250 s. PPP1R1B is highly expressed in the basal ganglia, including the lateral pallial olfactory area (LPO), but shows low expression in the neostriatum of the avian forebrain (Schnabel et al. 1997). These correspond to the medial striatum (MSt) and the nidopallium (NIC), respectively, according to the chicken brain atlas (Puelles et al. 2018). Based on this expression pattern, we determined the boundaries between these regions by identifying PPP1R1B-positive (stained) and -negative (unstained) areas. However, the boundaries between MSt and neighboring regions such as the lateral striatum (LSt), the pallial–subpallial transition zone (StPal), and the tubercular striatum (TuSt) were less clear. Therefore, to minimize contamination from adjacent areas, we quantified PPP1R1B-positive cells specifically in the mediodorsal part of the stained area, which most reliably corresponds to the MSt. The number of DARPP-32-positive neurons was calculated from monochrome photos of four parts of MSt (dorsomedial, dorsolateral, ventromedial, and ventrolateral) with 20× objectives. The photos were cut to a size of 500 µm × 500 µm, and the clipping images were used to calculate the number of immunoreactive cells (BZ-X800 Analyzer, Keyence).

In addition, the specificity of the antibody was verified in a preabsorption test when the antibody in a working dilution was incubated overnight at 4 °C with the antigen peptide used in antibody production (NH_2_-DPKDRKKIQFSVPAPPSQLDPR-COOH). The antigen–antibody mixture was used as the primary antiserum in the immunohistochemical procedure described above. The results showed that preabsorption eliminated staining (Fig. S5).

### Quantitative PCR

To confirm that the intramuscularly administered haloperidol affects *PPP1R1B* expression in the brain in the behavioral pharmacology test, the gene expression of *PPP1R1B* in the brain was quantified after administration of haloperidol. Male offensive-type chickens at 8 weeks of age received intramuscular administration of haloperidol decanoate or saline. Similar to RNA-sequencing, brain samples were collected and total RNA was prepared. Reverse transcription of total RNA (200 ng) was performed using the ReverTra Ace qPCR RT Kit (Toyobo). Samples contained SYBR Premix Ex Taq II (Takara Bio), 0.4 μM gene-specific primers (Table S5) and 2 µl synthesized cDNA in a 20 µl volume. qPCR was performed on a StepOnePlus Real-Time PCR System (Applied Biosystems) as follows: 95 °C for 20 s, followed by 40 cycles of 95 °C for 3 s and 60 °C for 30 s. The housekeeping gene *GAPDH* was used as an internal control, and no significant differences were found in *GAPDH* expression between the treatment.

### Statistical analysis

For behavioral and qPCR data, ANOVA was used to evaluate two or more factors, followed by post hoc multiple comparisons using the Tukey–Kramer's test. A Student's *t*-test was used to compare the two groups. Data were analyzed using the statistical software program Statcel2 (Figs. 1 and 4 and S1, S3, and S4). For immunohistochemistry data, a generalized linear mixed model was used in R (Fig. 3). The number of positive cells was treated as a response variable, group as a fixed effect, and position (eg dorsomedial) as a random effect.

## Supplementary Material

msag007_Supplementary_Data

## Data Availability

DNA-seq and RNA-seq data are available in the DDBJ BioProject database (accession number PRJDB16595). Codes for this study are archived in the GitHub repository: https://github.com/yypacific/UH_script.
