# Supplementary material for: Genomic and neurobiological bases of variation in fighting strategies in gamecocks
Source: Mol Biol Evol. 2026 Jan 9;43(1):msag007. doi: 10.1093/molbev/msag007 (PMC12835820; doi:10.1093/molbev/msag007)
Supplement: msag007_Supplementary_Data [file msag007_supplementary_data.zip › manuscript_22_MBE_revise_SupplFig.pdf]

Supplementary Information

Supplementary Figures

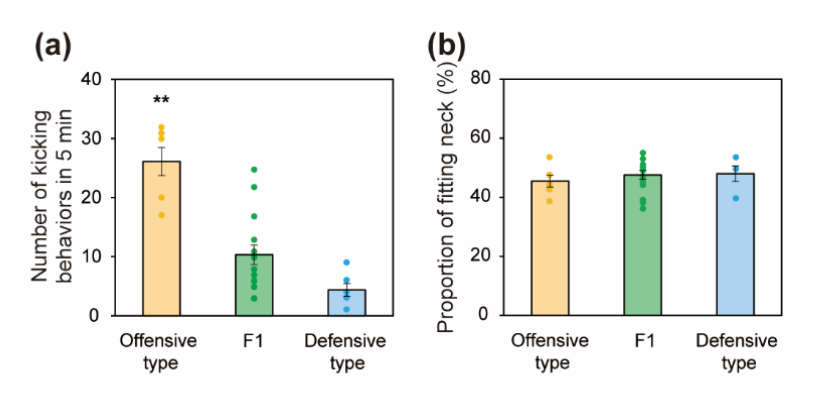

supplementary fig. S1. The crossbreeding experiment indicates that behavioral type at attack is genetically controlled. (a) F1 hybrids crossed the offensive and defensive type showed medium extent of offensive kicking behavior when the fought against defensive type ($^{**}P$ < 0.01, ANOVA, Tukey-Kramer's test; mean ± SEM, N=6-15). (b) Since the behavioral test was performed using defensive type as opponent, there is no significant difference in proportion of fitting neck (P = 0.723, ANOVA; mean ± SEM, N=6-15).

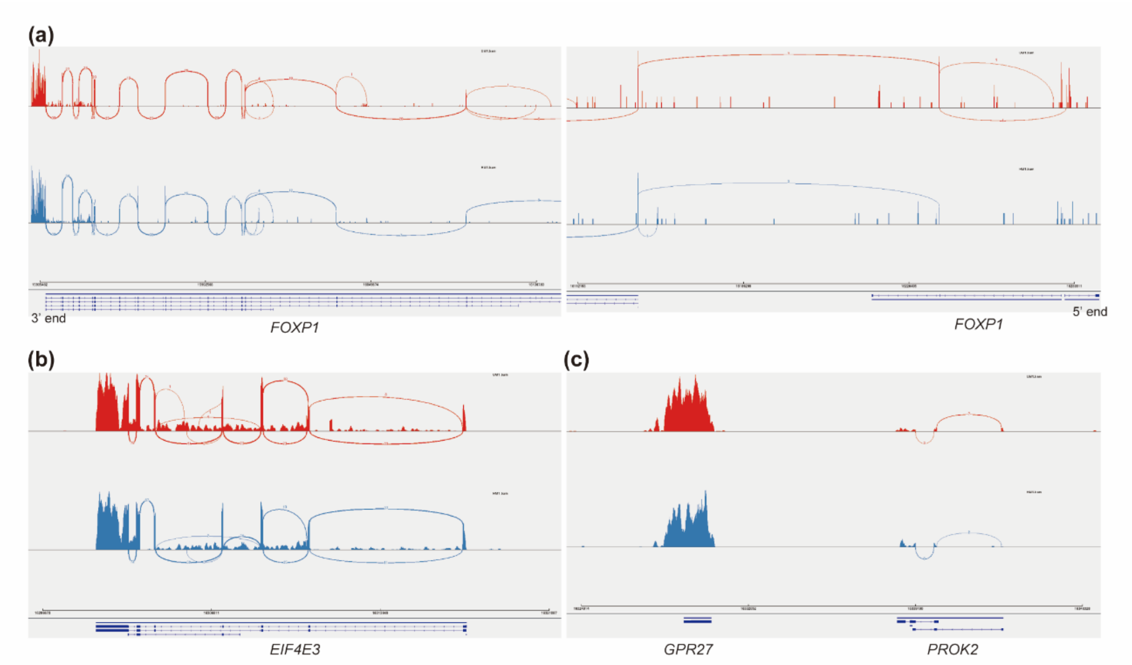

supplementary fig. S2. No significant differences of splicing were found between offensive and defensive type in the candidate region on chr. 12. (a-c) The representative sashimi plot showing the number of splicing junctions based on the RNA-seq data by Integrative Genome Viewer (IGV) indicated no significant difference between offensive (red) and defensive type (blue) in *FOXP1* (a), *EIF4E3* (b), *GPR27* and *PROK2* (**c**).

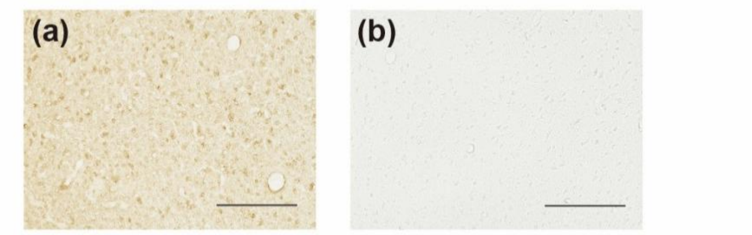

supplementary fig. S3. Brain images of immunohistochemistry (a) and pre-absorption test (b). Both images were obtained from the same subject (defensive type). The image in (a) is identical to Fig.3 (f). scale bar: 200 µm.

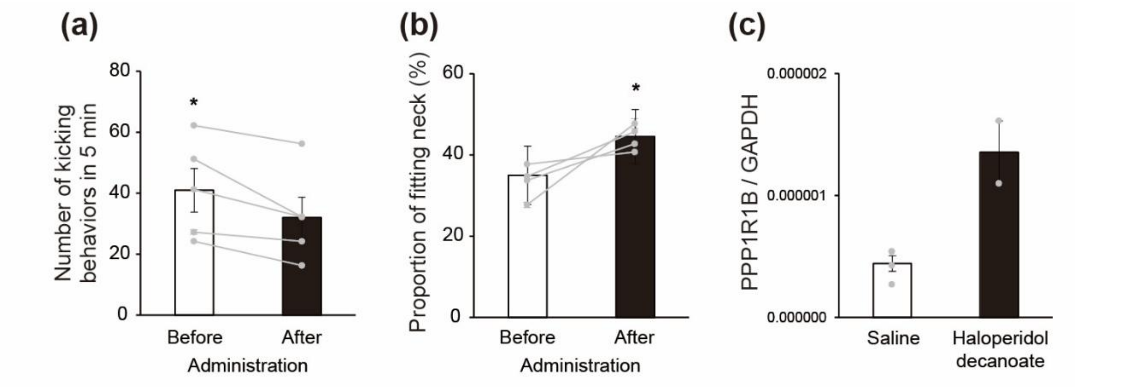

supplementary fig. S4. Imbalance of brain motor circuits results in different attack patterns. (a, b) Similarly with Figure 4a, the kicking behavior (a) was significantly decreased while fitting behavior (b) was increased in offensive type administrated before than 3 days after haloperidol decanoate ($^{*}P < 0.05$, $^{*}t$-test; mean ± SEM, N=5). (c) The *PPP1R1B* expression in diencephalon was higher in offensive type received haloperidol decanoate than saline (N=2-4).

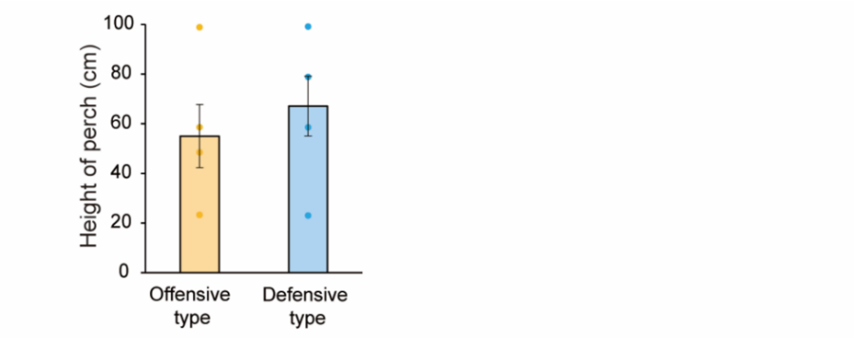

supplementary fig. S5. Defensive type has similar ability to jump up to the perch with offensive type. No significant difference between offensive and defensive type was found in the maximum height of perch that hens perched ($P = 0.501$, t-test; mean ± SEM, N=7 per type).

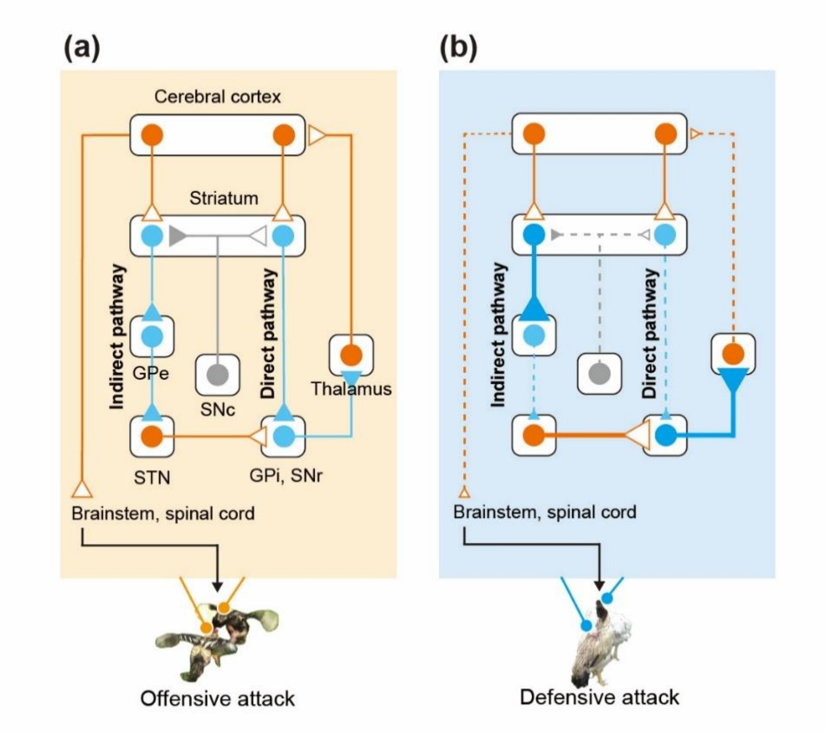

supplementary fig. S6. Possible mechanism underlying fighting strategies. The movement of animals is controlled by two circuits in the striatum: a direct pathway facilitating movement and an indirect pathway inhibiting movement. Specifically, direct pathway spiny projection neurons (dSPNs) project to the globus pallidus internal (GPi), whereas iSPNs project to the globus pallidus external (GPe). Ultimately, these pathways work to bidirectionally modulate excitatory input back into the cortex (Gerfen and Surmeier 2011; Cui, et al. 2013; Calabresi, et al. 2014). (a) Schematic figure of brain motor circuits of the offensive type whose direct and indirect pathways are normally cooperative. (b) In the defensive type, imbalance of the motor circuit by activation of an indirect pathway was suggested to result in a defensive attack. Light blue indicates GABAergic projection (inhibitory), red indicates glutamatergic projection (excitatory), and grey indicates dopaminergic projection (excitatory). GPe, globus pallidus external; GPi, globus pallidus internal; SNc, substantia nigra pars compacta; SNr, substantia nigra pars reticulata; STN, subthalamic nucleus.
